# Methodological review of the level of statistical support declared in radiological research articles

**DOI:** 10.1093/bjr/tqag026

**Published:** 2026-04-09

**Authors:** Amisha Pradhan, Tom Parry, Sue Mallett, Steve Halligan

**Affiliations:** Centre for Medical Imaging, University College London UCL, London WC1E 6JF, United Kingdom; Centre for Medical Imaging, University College London UCL, London WC1E 6JF, United Kingdom; Centre for Medical Imaging, University College London UCL, London WC1E 6JF, United Kingdom; Centre for Medical Imaging, University College London UCL, London WC1E 6JF, United Kingdom

**Keywords:** statistical data analysis, meta-research, research design, methods

## Abstract

**Objectives:**

We assessed if there was disparity between qualified statisticians and other researchers regarding the level of statistical assistance deemed necessary to support radiological research.

**Methods:**

We categorized 50 consecutive, eligible original research articles published in an indexed imaging journal (*European Radiology*) in 2024, according to authors’ statements regarding statistical support, declared in the “Statistics and Biometry” section. Two reviewers extracted data related to study design, statistical methods, and analysis. Two medical statisticians categorized each study as presenting “complex” statistical methods or not and then compared this with authors’ own assessment of statistical complexity, stated in the published article. We performed descriptive analyses.

**Results:**

Most studies were observational (49, 98%) and retrospective (38, 76%). 35 (70%) studies were diagnostic, 7 (14%) prognostic, and 6 (12%) mixed. Malignancy was the most frequent topic (29 studies, 58%), and MRI the most frequent modality (35 studies, 70%). We deemed most studies (33, 66%) presented complex statistical methods. Of these, 13 studies (26% overall) declared that “no complex statistical methods were necessary for this paper.” However, 10 of these employed hypothesis testing, frequently using multiple methods; 9 employed agreement and/or reliability analyses; all presented accuracy measures; 11 (85%) presented a regression model.

**Conclusions:**

We found that approximately one quarter of original research articles published in our sample stated that “no complex statistical methods were necessary,” but then presented complex analyses.

**Advances in knowledge:**

Some radiological researchers may underestimate the complexities of statistical analysis and requirement for specialist statistical support, which risks inappropriate analyses and misleading results.

## Introduction

Clinical research is fundamental to advance medical practice, and requires sound study hypotheses, design, analysis, and interpretation. However, concerns have been raised repeatedly regarding the poor methodological quality of medical research in general.^1–3^ Indeed, some estimates suggest that around 85% of health research is fundamentally flawed.^4^ Imaging research is not exempt; one study found that 147 (94%) of 157 research articles published in radiology journals contained at least one statistical error.^5^ Inability to access appropriate statistical support undoubtedly encourages poor study design and analysis; a recent study revealed that radiology trainees were expected to “do research” without statistical support.^6^ While it takes several years to train a medical statistician, many clinicians tackle study design and analysis with little or no training.^7^ This has potentially serious consequences because acting on incorrect research findings risks harming patients^1^^,^^8^^,^^9^

*European Radiology* is an indexed, peer-reviewed journal, that obliges researchers to declare the level of statistical support provided, achieved via a “disclosure paragraph” that is published alongside accepted articles. This presents the opportunity to assess Authors’ judgments regarding need for statistical assistance. We hypothesized there may be disparity between qualified statisticians and other researchers regarding the level of statistical provision deemed necessary to support the published research. To examine this, we asked qualified medical statisticians to assess the statistical complexity of research articles published in European Radiology, and then compared their assessments with the level of complexity declared by the authors. We aimed to determine what proportion, if any, of radiological researchers apparently underestimate statistical complexity.

## Methods

Ethical permission is not required by our institution for review of primary literature. We developed a protocol prior to data extraction.

### Information sources and search string

Via the PubMed database, a single researcher (AP) searched the National Library of Medicine Catalog of indexed journals, for original research articles published in *European Radiology* over a 6-month time horizon (May 2024 to October 2024 inclusive). For this methodological review we elected to analyse a representative sample (as opposed to a full dataset) and hypothesized that 50 eligible articles would provide sufficient material to achieve data saturation (ie, the point at which additional data provides no new insight). The search string excluded publications unlikely to contain statistical analysis and is detailed in Supplementary Material S1. Selection was based on progressively ascending issue date to facilitate sample size expansion if data were insufficient (versus selection by random sampling or stratification).

### Study eligibility and selection

Two reviewers (AP and TP), one of whom was a medical statistician, then independently screened article titles and abstracts for eligibility, defined by potential for statistical analysis, ie, case-control, cross-sectional, and cohort studies; clinical trials; systematic reviews (with or without meta-analysis). Narrative reviews, editorials, commentary, and correspondence were excluded if encountered. The full article was then obtained for potentially eligible articles. These were searched for statistical analysis and excluded if none was present. Two medical statisticians (TP and SM) defined statistical analysis as any summary statistic beyond those described by “baseline” characteristics (often found in “Table 1” of results from typical articles).

**Table 1:** Statistical analysis categorization table to facilitate consistency of data classification during extraction.

Type of statistical analysis:	
Accuracy	Sensitivity/specificity ROC-AUC Net benefit
Hypothesis testing	*t*-test ANOVA Chi-squared Fisher’s exact test Two-proportions Z-test Wilcoxon rank
Regression	Univariable Multivariable Linear Logistic Other
Repeatability (agreement or reliability)	Percentage agreement Bland-Altman Pearson correlation coefficient Spearman’s rank ICC Cohen’s *d* Cohen’s kappa Cronbach’s alpha Kendall’s tau

### Data extraction

Extraction was piloted on 10 studies outside the search dates. (AP) then extracted the following data from individual studies: target condition; subject; temporal design (prospective/retrospective); study type and phase (eg, observational, interventional [ie, therapeutic intervention], systematic review, diagnostic and/or prognostic); imaging modality; primary outcome statement; missing data statement; sample size calculation; statistical analysis type (descriptive, hypothesis testing, agreement/reliability measure, accuracy, regression analysis, survival analysis etc.); statistical software used.

The “Ethics declarations” section concluding each article was consulted and the “statistics and biometry” response was identified from the available categories, as follows: “XXX kindly provided statistical advice for this manuscript”; “One of the authors has significant statistical expertise”; “no complex statistical methods were necessary for this paper.”

*European Radiology* does not define “complex statistical methods,” nor are we aware of any existing definition elsewhere. Therefore, prior to extraction two medical statisticians (TP and SM) defined “complex” as any method that required an understanding of statistical concepts and methods beyond those taught commonly at medical school and understood by most with medical knowledge (eg, mean, median, standard deviation, simple hypothesis tests [*t*-test, chi-squared], simple correlation). For example, aspects influencing perception of complexity included whether outputs were difficult to interpret without formal statistical training and/or whether custom software coding was desirable to execute statistical methods (as opposed to “point and click” analyses). To facilitate extraction, the two statisticians compiled a statistical test categorization table to ensure consistency of data classification during extraction (Table 1). The two reviewers (AP and TP) reviewed the entire text of each article independently, with a focus on the methodology and results sections, which detail statistical methods. They then categorized each article as describing “complex” statistical methods or not or, where necessary, noted that statistical methods were not appropriate for the article. Any discrepancy was resolved by discussion with a professor of medical statistics (SM).

All data were extracted into a spreadsheet (Excel, Microsoft Corporation, Redmond, USA). Any discrepancy was resolved by face-to-face discussion, involving senior authors (SM and SH) where necessary.

### Analysis

We performed descriptive analysis summarizing review findings as median, interquartile range (IQR), and range.

## Results

**Figure 1:**
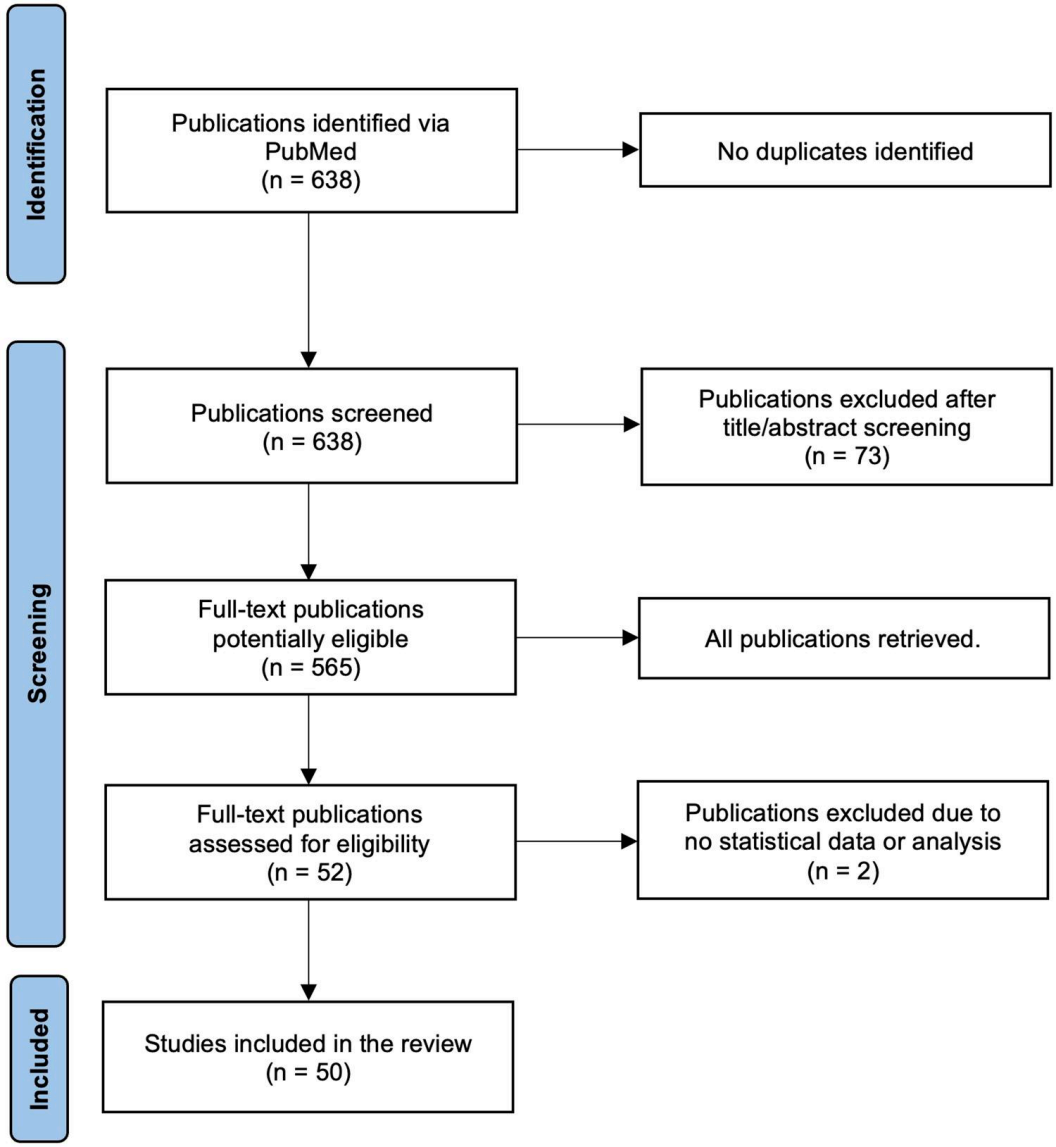
Flow chart of studies selected for the review.

The search string identified 638 articles, without duplicates. After screening, 565 of these were searched consecutively (starting from the oldest), until 50 had been selected for inclusion; the study flow diagram is illustrated in Figure 1. Included studies are shown in Material S2. Study characteristics are shown in Table 2. Studies originated from 13 individual countries, with China (22 studies, 44% from 15 universities; 3 universities contributed 5, 3, and 2 articles respectively), South Korea (8 studies, 16% from 5 universities; 2 universities contributed 3 and 2 studies each), and Germany (5, 10%) the most frequent. The very large majority were observational studies (49, 98%) and most were retrospective (38, 76%), with a single systematic review.^10^ Most studies were diagnostic (35, 70%), with 7 (14%) prognostic and 6 (12%) both prognostic and diagnostic. Two studies were interventional. All study subjects were human, with mean sample size 244, range 35^11^ to 19 581,^12^ IQR 216.75. Malignant neoplasms were the most frequent topic (29, 58%), followed by neurological conditions (4, 8%). The most frequent modality was MRI (35, 70%), followed by CT (16, 32%).

**Table 2:** Characteristics of studies included in the review.

Study	Country	Target condition	Subject type	Temporal design	Study phase	Study type	Type of imaging data	Highest number of patients/specimens in Table 1
Rosen and Saban (2024)	Israel	N/A	Human	Retrospective	Observational	Diagnostic	CT	97
Lee et al. (2024b)	South Korea	Malignant neoplasms	Human	Prospective	Observational	Diagnostic and prognostic	MRI CT	71
Que et al. (2024a)	China	Malignant neoplasms	Human	Retrospective	Observational	Diagnostic and prognostic	MRI	91
Qu et al. (2024b)	China	Neurological conditions	Human	Retrospective	Observational	Diagnostic	MRI	159
Xia et al. (2024)	United States	Musculoskeletal diseases	Human	Prospective	Observational	Diagnostic	MRI	43
Lee et al. (2024a)	South Korea	Genitourinary diseases	Human	Retrospective	Observational	Interventional	US MRI	180
Xie et al. (2024)	China	Musculoskeletal diseases	Human	Retrospective	Observational	Diagnostic	X-ray	3823
Park et al. (2024b)	South Korea	Malignant neoplasms	Human	Retrospective	Observational	Diagnostic	MRI	103
Sim et al. (2024)	South Korea	Infectious and parasitic diseases	Human	Retrospective	Observational	Diagnostic	MRI	154
Heo et al. (2024)	South Korea	Malignant neoplasms	Human	Retrospective	Observational	Prognostic	MRI CT	204
Yan et al. (2024)	China	Neurological conditions	Human	Retrospective	Observational	Diagnostic	MRI	135
Wang et al. (2024a)	China	Malignant neoplasms	Human	Retrospective	Observational	Diagnostic	MRI X-ray	98
Zhang et al. (2024)	China	Malignant neoplasms	Human	Prospective	Observational	Diagnostic	MRI	65
Chen et al. (2024)	China	Malignant neoplasms	Human	Retrospective	Observational	Diagnostic	MRI	1192
Wu et al. (2024b)	China	Malignant neoplasms	Human	Prospective	Observational	Diagnostic	MRI	129
Wang et al. (2024c)	China	Unintentional injuries	Human	Prospective	Observational	Diagnostic	US	96
Waelti et al. (2024)	Switzerland	N/A	Human	Retrospective	Observational	Diagnostic	PET CT MRI	243
Marth et al. (2024)	Switzerland	Musculoskeletal diseases	Human	Retrospective	Observational	Diagnostic	MRI	168
Schwarting et al. (2024)	Germany	Oral conditions	Human	Prospective	Observational	Diagnostic and prognostic	MRI	35
Quan et al. (2024)	China	Neurological conditions	Human	Retrospective	Observational	Prognostic	MRI	274
Rigiroli et al. (2024)	United States	Malignant neoplasms	Human	Retrospective	Observational	Diagnostic	CT	828
Zhou et al. (2024)	China	Cardiovascular diseases	Human	Prospective	Observational	Diagnostic	MRI	74
Lee et al. (2024c)	South Korea	Malignant neoplasms	Human	Retrospective	Observational	Diagnostic and prognostic	CT	3181
Lv et al. (2024)	China	Cardiovascular diseases	Human	Prospective	Observational	Diagnostic and prognostic	MRI	132
Jiang et al. (2024)	China	Malignant neoplasms	Human	Retrospective	Observational	Prognostic	MRI	600
Peters et al. (2024)	Germany	Malignant neoplasms	Human	Retrospective	Observational	Diagnostic	CT	169
Yu et al. (2024)	China	Malignant neoplasms	Human	Retrospective	Observational	Interventional	CT MRI	222
Matsumoto et al. (2024)	Japan	Malignant neoplasms	Human	Retrospective	Observational	Diagnostic	CT MRI	364
Sanvito et al. (2024)	United States	Malignant neoplasms	Human	Prospective	Observational	Diagnostic	MRI	38
Nakai et al. (2024)	United States	Malignant neoplasms	Human	Retrospective	Observational	Diagnostic	MRI	11319
Wang et al. (2024b)	China	Cardiovascular diseases	Human	Retrospective	Observational	Prognostic	MRI	314
He et al. (2024)	China	Neurological conditions	Human	Retrospective	Observational	Diagnostic	PET MRI	1026
Wu et al. (2024a)	China	Malignant neoplasms	Human	Retrospective	Observational	Diagnostic and prognostic	MRI	77
Yun et al. (2024)	South Korea	Malignant neoplasms	Human	Retrospective	Observational	Diagnostic	MRI	310
Bilreiro et al. (2024)	Portugal	Malignant neoplasms	Human	N/A	Systematic review &/or meta-analysis	Diagnostic	MRI	161
Chang et al. (2024)	Taiwan	Malignant neoplasms	Human	Retrospective	Observational	Diagnostic	CT	242
Jacques et al. (2024)	France	Unintentional injuries	Human	Retrospective	Observational	Diagnostic	CT X-ray	296
Loch et al. (2024)	Germany	Malignant neoplasms	Human	Retrospective	Observational	Diagnostic	CT	91
Gertz et al. (2024)	Germany	Cardiovascular diseases	Human	Retrospective	Observational	Diagnostic	CT	131
Kerpel-Fronius et al. (2024)	Hungary	Malignant neoplasms	Human	Prospective	Observational	Diagnostic	CT	4215
Frood et al. (2024)	United Kingdom	Malignant neoplasms	Human	Retrospective	Observational	Prognostic	PET CT	444
Kong et al. (2024)	China	Malignant neoplasms	Human	Retrospective	Observational	Diagnostic	CT	78
Xiao et al. (2024)	China	Malignant neoplasms	Human	Retrospective	Observational	Diagnostic	MRI	117
Park et al. (2024a)	South Korea	Malignant neoplasms	Human	Retrospective	Observational	Prognostic	MRI	68
Xu et al. (2024)	China	Malignant neoplasms	Human	Retrospective	Observational	Prognostic	MRI	284
Crimì et al. (2024)	Italy	Malignant neoplasms	Human	Retrospective	Observational	Diagnostic	MRI	139
Li et al. (2024)	China	Respiratory infectious	Human	Retrospective	Observational	Diagnostic	X-ray	4400
Zuiani et al. (2024)	Italy	Malignant neoplasms	Human	Retrospective	Observational	Diagnostic	MRI	201
Ni et al. (2024)	China	Unintentional injuries	Human	Prospective	Observational	Diagnostic	MRI	68
Nowak et al. (2024)	Germany	N/A	Human	Retrospective	Observational	Diagnostic	X-ray	19,581

See online supplementary material for full study references.

Table 3 details the level of statistical support declared by the 50 studies, compared with our own statisticians’ assessment of whether formal statistical support was necessary or not. We deemed support was necessary for most studies (33, 66%). Of these, while authors stated that statistical advice was provided for 4, and that 1 of the authors had significant statistical expertise in a further 16, 13 studies (26% of total) declared that no complex statistical methods were necessary. We deemed specific statistical support was unnecessary for 17 studies (34% overall). As expected, none of these studies declared explicitly that external statistical advice had been provided: 9 stated that no complex statistical methods were necessary and the remaining 8 stated that 1 of the authors had significant statistical expertise.

**Table 3:** Level of statistical support declared for 50 research studies, compared with whether support was deemed necessary.

Level of statistical support declared:	Statistical support deemed necessary, *n* (%)	Statistical support deemed unnecessary, *n* (%)	Total (*n = *50)
“No complex statistical methods were necessary”	13 (26)	9 (18)	22 (44)
“One of the authors has significant statistical expertise”	16 (32)	8 (16)	24 (48)
“XX kindly provided statistical advice”	4 (8)	0 (0)	4 (8)
Total	33 (66%)	17 (34%)	

The presence of all 7 categories of statistical methods was assessed across all studies. Table 4 details these categories, split by whether these were present or not in the 50 articles selected for the review. Table 4 also splits the data according to whether statistical support was used or not, and whether we deemed statistical support was necessary or not. Descriptive analysis was the commonest method (absent in only 1 study), followed by hypothesis testing (44 studies, 88%), accuracy analysis (36, 72%), regression analysis (30, 60%), repeatability analysis (28, 56%), and survival analysis (11, 22%). Sample size calculation was found least frequently (4, 8%; deemed unnecessary for 2 studies).^11^^,^^13–15^ A primary outcome was stated explicitly by just 5 studies (10%).^16–20^ Only 11 studies (22%) described how missing data were handled statistically (missing data were not applicable to 1 study).

**Table 4:** Table indicating 7 individual categories of statistical method, categorized by whether these were present in the 50 articles selected for the review.

Statistical method	Statistical support utilized	Statistical support deemed necessary but not used (*n = *13)	Statistical support deemed necessary and used (*n = *20)	Statistical support deemed unnecessary and not used (*n = *9)	Statistical support deemed unnecessary but used (*n = *8)	Total
Sample size calculation	Not applicable	0 (0%)	0 (0%)	1 (11%)	1 (12%)	2 (4%)
	Yes	1 (8%)	1 (5%)	1 (11%)	1 (12%)	4 (8%)
	No	12 (92%)	19 (95%)	7 (78%)	6 (75%)	44 (88%)
Descriptive analysis	Yes	12 (92%)	20 (100%)	9 (100%)	8 (100%)	49 (98%)
	No	1 (8%)	0 (0%)	0 (0%)	0 (0%)	1 (2%)
Hypothesis testing	Yes	10 (77%)	18 (90%)	8 (89%)	8 (100%)	44 (88%)
	No	3 (23%)	2 (10%)	1 (11%)	0 (0%)	6 (12%)
Repeatability analysis	Yes	9 (69%)	11 (55%)	4 (44%)	4 (50%)	28 (56%)
	No	4 (31%)	9 (45%)	5 (56%)	4 (50%)	22 (44%)
Accuracy analysis	Yes	13 (100%)	14 (70%)	6 (67%)	3 (38%)	36 (72%)
	No	0 (0%)	6 (30%)	3 (33%)	5 (62%)	14 (28%)
Regression analysis	Yes	11 (85%)	17 (85%)	2 (22%)	0 (0%)	30 (60%)
	No	2 (15%)	3 (15%)	7 (78%)	8 (100%)	20 (40%)
Survival analysis	Not applicable	9 (69%)	9 (45%)	5 (56%)	8 (100%)	31 (62%)
	Yes	4 (31%)	7 (35%)	0 (0%)	0 (0%)	11 (22%)
	No	0 (0%)	4 (20%)	4 (44%)	0 (0%)	8 (16%)

Articles have been split by whether statistical support was used or not, and whether statistical support was deemed necessary following our review or not.

Table 5 details the 13 studies where the authors stated, “no complex statistical methods were necessary for this paper,” but where our statisticians deemed complex methods were present in the manuscript.^12^^,^^14^^,^^17^^,^^21–30^ The large majority (11, 85%) were retrospective, with 9 (69%) solely diagnostic, 2 solely prognostic, and the remaining 2 mixed. Most studies (10, 77%) employed hypothesis testing, frequently incorporating multiple methods within a single study. Nine studies (69%) employed agreement and/or reliability analyses, and all presented accuracy measures. Most studies (11, 85%) presented a regression model, with 4 incorporating survival analysis.

**Table 5:** Thirteen studies assessed as requiring complex statistical methods but where authors’ deemed this unnecessary.

Study	Temporal design	Study design	Hypothesis test	Agreement or reliability measure	Accuracy measure	Regression model	Survival analysis
Que et al^21^	Retrospective	Diagnostic & prognostic	Log-rank	Cohen’s kappa	AUC, sensitivity, specificity	Cox	Yes
Qu et al^22^	Retrospective	Diagnostic	N/A	Bland-Altman	AUC, sensitivity, specificity	Machine learning	N/A
Park et al^23^	Retrospective	Diagnostic	Chi-squared, *t*-test	N/A	AUC, sensitivity, specificity, Youden’s index	Logistic	N/A
Yan *et al*^24^	Retrospective	Diagnostic	Chi-squared, Mann-Whitney, Anova, Ancova, DeLong	Unknown	AUC	Logistic	N/A
Zhang *et al*^14^	Prospective	Diagnostic	*t*-test, Shapiro-Wilk	N/A	AUC, sensitivity, specificity, net-benefit	Logistic	N/A
Chen *et al*^25^	Retrospective	Diagnostic	N/A	N/A	AUC, sensitivity, specificity, F-score	Machine learning	N/A
Lv et al^17^	Prospective	Diagnostic & prognostic	*t*-test, Chi-square, Fisher’s Exact	ICC	AUC	Cox	Yes
Wang et al^26^	Retrospective	Prognostic	Shapiro-Wilk, *t*-test, Wilcoxon signed rank, De Long’s log-rank, Schoenfield residuals	Bland-Altman	AUC	Linear Cox	Yes
Chang et al^27^	Retrospective	Diagnostic	Kruskall-Wallis, Chi-Squared, Mann-Whitney	Cohen’s Kappa	Sensitivity, specificity, PPV, NPV, accuracy, Youden’s Index	Logistic	N/A
Kong et al^28^	Retrospective	Diagnostic	Chi-squared, Fisher’s exact, *t*-test, Wilcoxon signed-rank	N/A	AUC, sensitivity, specificity, PPV, accuracy,	N/A	N/A
Xu et al^29^	Retrospective	Prognostic	*t*-test, log rank	Weighted Kappa, Pearson’s correlation	AUC, sensitivity, specificity	Cox	Yes
Li et al^30^	Retrospective	Diagnostic	Wilcoxon signed-rank	Dice-Sorensen	AUC, sensitivity, specificity, accuracy, F-score	Machine learning	N/A
Nowak et al^12^	Retrospective	Diagnostic	N/A	Cohen’s Kappa	AUC, sensitivity, specificity	N/A	N/A

All studies were observational.

Abbreviations: AUC = area under the receiver-operator curve; ICC = intra-class correlation; N/A = not applicable.

## Discussion

Using experienced medical statisticians, we assessed the statistical complexity of research articles published in *European Radiology* and then compared our assessment with Authors’ own. We wished specifically to determine if any articles stated that “no complex statistical methods were necessary” but where the methods presented appeared to contradict this. Ultimately, this applied to around a quarter of our sample. The obvious interpretation is that some radiological researchers underestimate statistical difficulty, tackling complex analyses themselves. It is self-evident that unreliable results may arise when untrained researchers attempt analyses more appropriate for trained statisticians. Systematic review has established that studies involving statisticians present fewer design and analysis flaws, and present more accurate analytical methods than those that do not.^31^

Proper statistical analysis demands far more than simply inputting data, choosing a test, and then executing a command. Rather, statisticians understand which analyses best address the research question to inform clinical practice. They know which tests suit the data structure (and which are contraindicated), can account for potential confounders and, perhaps most importantly, can advise whether analysis is even appropriate due to inadequate power or poor design. Indeed, statisticians are best consulted early, to help frame the research question precisely, choose an appropriate design, and help define the endpoints and participant numbers necessary for the results to provide good evidence.^31^ Statisticians can also help with unbiased interpretation. However, a systematic review found that 61% of medical researchers believed their own statistical skills sufficiently advanced to negate the need for a statistician.^31^ Nevertheless, “experience” of analysis is quite distinct from “expertise.” It is exceedingly easy for an untrained radiological researcher to unwittingly employ an inappropriate method, produce misleading results, and then interpret these inaccurately.^32^ For example, a recent systematic review of studies in Crohn’s disease found that authors frequently failed to differentiate between diagnostic and prognostic data and often combined the two inadvertently.^33^ In the present study, we found studies claiming “no complex methods were necessary” but which used survival analysis inappropriately to answer a diagnostic rather than prognostic question. Of the 13 studies declaring that no complex statistical methods were necessary and which were deemed to present complex statistical methods, 3 developed multivariable prognostic models. Each selected predictors for the multivariable model from those achieving *P* < .1 in univariable analysis. This data-driven approach risks overfitting, ignores confounding, and reduces the clinical relevance of the model. Only 1 of the 3 studies considered the proportional hazards assumption, but, even then, relied on a *P*-value from statistical testing rather than clinical judgment, again risking overfitting.

Of course, whether a particular statistical analysis is “complex” or not may be a matter of opinion. Irrespective, this matter is clearly best considered by trained statisticians rather than radiologists, which is why we used medical statisticians to categorize analytical complexity. While it is obvious that a need for in-house coding indicates complexity (ie, the method is not generally available in statistical analysis packages or the data structure is sufficiently complex to require custom coding), we considered “point & click” software analyses with great caution because, while execution may be simple, the underlying assumptions and analysis may be complex. We categorized the type of statistical analyses encountered (Table 1) and initially intended to use this to simultaneously categorize method complexity. However, we found this was not possible because methodological complexity is highly dependent upon the individual study scenario and data structure. Accordingly, our statisticians were obliged to assess individual papers in great detail.

For example, “regression” can be linear or logistic, univariable or multivariable. A recent systematic review of highly indexed radiological journals found that over half of published research articles presented a model, usually multivariable, most did not include a statistician, and the very large majority did not cite the relevant reporting guideline.^34^ Our present review found that 85% of the articles considered complex by us but not by the authors, presented a regression model. In contrast, statisticians generally regard multivariable modelling as complex. Considerable statistical skill is required to avoid multiple analyses that generate excessive false positive results, and to then interpret multivariable regression coefficients without “Table 2 fallacy,” a very common error.^35–38^

While *European Radiology* provides no definition of “significant statistical expertise,” we anticipate this would be difficult to define. Interestingly, there is some disagreement amongst statisticians themselves regarding exactly what constitutes a “qualified” medical statistician.^31^ While a degree in medical statistics would be widely regarded as appropriate, there are other routes to competence (eg, formal training in health technology assessment). Conversely, some individuals with seemingly appropriate qualifications may not be expert because, like medicine, statisticians often have subspecialist interests that may render them unable to tackle all statistical issues. One of our radiologist authors (SH) has a PhD in medical statistics but cannot be considered a qualified statistician.

Our study does have limitations. In particular, our research was confined to a single journal because we were aware that *European Radiology* obliges authors to declare the level of statistical report provided. Like most imaging journals we submit to, the *British Journal of Radiology* does not require any specific details regarding the statistical support used for the research, and so similar research would not be possible. However, we have no reason to expect our findings would not generalize to similar journals. We were obliged to rely on the classifications stipulated by *European Radiology*. For example, the statement, “XXX kindly provided statistical advice for this manuscript” does not require that the individual in question must be a qualified statistician. Also, as noted above, “significant statistical expertise” is undefined. It follows that where statistical support was obtained, it was not possible to determine the exact quality and level of this for all individual articles. One of our statisticians (TP) performed an informal author search for some articles that stated, “One of the authors has significant statistical expertise,” and was reassured that the majority appeared linked to statistical or methodological departments rather than radiological. In any event, these issues do not affect our primary outcome, which was based on articles stating that, “no complex statistical methods were necessary for this paper.” Journals that oblige authors to state their level of statistical support may wish to consider modifying and/or expanding their definitions so that there is more certainty regarding exactly what level of support was provided, in particular whether obtained from a qualified statistician or not. We did not investigate any association between country of origin and level of statistical support declared, which would require a much larger sample size to ensure sufficient component countries to power an analysis adequately. Similarly, we did not stratify by country of origin, meaning that some are over-represented in our sample; Notably China contributed 44% and South Korea 16%. Given selection was consecutive, we assume this reflects the range and proportion of countries publishing research currently. For similar reasons we did not intend an institutional analysis but note that 5 institutions (3 from China and 2 from South Korea) contributed multiple articles to our sample, which might introduce a cluster bias. While it is possible that an institution with particularly poor statistical support might encourage researchers to tackle complex analyses themselves, we would expect this to affect smaller rather than larger universities.

In summary, our review of published research articles found that, when assessed by independent medical statisticians, approximately one quarter of articles stating that “no complex statistical methods were necessary,” actually presented complex analyses. This suggests a significant proportion of radiological researchers underestimate statistical difficulty, tackling complex analyses themselves.

## Supplementary Material

tqag026_Supplementary_Data
