# Supplementary material for: Methodological review of the level of statistical support declared in radiological research articles
Source: Br J Radiol. 2026 Apr 9;99(1181):921–30. doi: 10.1093/bjr/tqag026 (PMC13134853; doi:10.1093/bjr/tqag026)
Supplement: tqag026_Supplementary_Data [file tqag026_supplementary_data.docx]

**Supplementary Material.**

**Electronic Supplementary Material 1:**

Search String (performed 14^th^ December 2024):

(((((((("european radiology"[Journal]) NOT ("comment"[Publication Type])) NOT

("corrected and republished article"[Publication Type])) NOT ("published

erratum"[Publication Type])) NOT ("editorial"[Publication Type])) NOT ("letter"[Publication

Type])) NOT ("news"[Publication Type])) NOT ("newspaper article"[Publication Type]))

AND (("2024/05"[Date - Publication] : "2024/10"[Date - Publication]))

**Electronic Supplementary Material 2:**

List of studies included in the review:

1. Bilreiro, C., Andrade, L., Marques, R. M. and Matos, C. (2024). Diffusion-weighted imaging for determining response to neoadjuvant therapy in pancreatic cancer: a systematic review and meta-analysis. *Eur Radiol,* 34**,** 3238-3248 doi: 10.1007/s00330-023-10381-0.
2. Chang, Y. C., Chen, P. T., Hsieh, M. S., Huang, Y. S., Ko, W. C., Lin, M. W., Hsu, H. H., Chen, J. S. and Chang, Y. C. (2024). Discrimination of invasive lung adenocarcinoma from Lung-RADS category 2 nonsolid nodules through visual assessment: a retrospective study. *Eur Radiol,* 34**,** 3453-3461 doi: 10.1007/s00330-023-10317-8.
3. Chen, J., Xue, Y., Ren, L., Lv, K., Du, P., Cheng, H., Sun, S., Hua, L., Xie, Q., Wu, R. and Gong, Y. (2024). Predicting meningioma grades and pathologic marker expression via deep learning. *Eur Radiol,* 34**,** 2997-3008 doi: 10.1007/s00330-023-10258-2.
4. Crimì, F., Cabrelle, G., Campi, C., Schillaci, A., Bao, Q. R., Pepe, A., Spolverato, G., Pucciarelli, S., Vernuccio, F. and Quaia, E. (2024). Nodal staging with MRI after neoadjuvant chemo-radiotherapy for locally advanced rectal cancer: a fast and reliable method. *Eur Radiol,* 34**,** 3205-3214 doi: 10.1007/s00330-023-10265-3.
5. Frood, R., Mercer, J., Brown, P., Appelt, A., Mistry, H., Kochhar, R. and Scarsbrook, A. (2024). Training and external validation of pre-treatment FDG PET-CT-based models for outcome prediction in anal squamous cell carcinoma. *Eur Radiol,* 34**,** 3194-3204 doi: 10.1007/s00330-023-10340-9.
6. Gertz, R. J., Gerhardt, F., Pienn, M., Lennartz, S., Kröger, J. R., Caldeira, L., Pennig, L., Schömig, T. H., Hokamp, N. G., Maintz, D., Rosenkranz, S. and Bunck, A. C. (2024). Dual-layer dual-energy CT-derived pulmonary perfusion for the differentiation of acute pulmonary embolism and chronic thromboembolic pulmonary hypertension. *Eur Radiol,* 34**,** 2944-2956 doi: 10.1007/s00330-023-10337-4.
7. He, K., Li, B., Huang, L., Zhao, J., Hua, F., Wang, T., Li, J., Wang, J., Huang, Q., Chen, K., Xu, S., Ren, S., Cai, H., Jiang, D., Hu, J., Han, X., Guan, Y., Chen, K., Guo, Q. and Xie, F. (2024). Positive rate and quantification of amyloid pathology with [(18)F]florbetapir in the urban Chinese population. *Eur Radiol,* 34**,** 3331-3341 doi:10.1007/s00330-023-10366-z.
8. Heo, S., Kang, H. J., Choi, S. H., Kim, S., Yoo, Y., Choi, W. M., Kim, S. Y. and Lee, S. S. (2024). Proliferative hepatocellular carcinomas in cirrhosis: patient outcomes of LI-RADS category 4/5 and category M. *Eur Radiol,* 34**,** 2974-2985 doi: 10.1007/s00330-023-10305-y.
9. Jacques, T., Cardot, N., Ventre, J., Demondion, X. and Cotten, A. (2024). Commercially available AI algorithm improves radiologists' sensitivity for wrist and hand fracture detection on X-ray, compared to a CT-based ground truth. *Eur Radiol,* 34**,** 2885-2894 doi: 10.1007/s00330-023-10380-1.
10. Jiang, H., Qin, Y., Wei, H., Zheng, T., Yang, T., Wu, Y., Ding, C., Chernyak, V., Ronot, M., Fowler, K. J., Chen, W., Bashir, M. R. and Song, B. (2024). Prognostic MRI features to predict postresection survivals for very early to intermediate stage hepatocellular carcinoma. *Eur Radiol,* 34**,** 3163-3182 doi: 10.1007/s00330-023-10279-x.
11. Kerpel-Fronius, A., Megyesfalvi, Z., Markóczy, Z., Solymosi, D., Csányi, P., Tisza, J., Kecskés, A., Baranyi, B., Csánky, E., Dóka, A., Gálffy, G., Göcző, K., Győry, C., Horváth, Z., Juhász, T., Kállai, Á., Kincses, Z. T., Király, Z., Király-Incze, E., Kostyál, L., Kovács, A., Kovács, A., Kuczkó, É., Makra, Z., Maurovich Horvát, P., Merth, G., Moldoványi, I., Müller, V., Pápai-Székely, Z., Papp, D., Polgár, C., Rózsa, P., Sárosi, V., Szalai, Z., Székely, A., Szuhács, M., Tárnoki, D., Tavaszi, G., Turóczi- Kirizs, R., Tóth, L., Urbán, L., Vaskó, A., Vigh, É., Dome, B. and Bogos, K. (2024). HUNCHEST-II contributes to a shift to earlier-stage lung cancer detection: final results of a nationwide screening program. *Eur Radiol,* 34**,** 3462-3470 doi: 10.1007/s00330-023-10379-8.
12. Kong, Z., Wang, J., Ni, S., Liu, Y., Zhao, X., Zhu, Y., Li, L. and Liu, S. (2024). CT-based quantification of trachea shape to detect invasion by thyroid cancer. *Eur Radiol,* 34**,** 3141-3150 doi: 10.1007/s00330-023-10301-2.
13. Lee, S., Lee, C. Y., Kim, N. Y., Suh, Y. J., Lee, H. J., Yong, H. S., Kim, H. R. and Kim, Y. J. (2024b). Feasibility of UTE-MRI-based radiomics model for prediction of histopathologic subtype of lung adenocarcinoma: in comparison with CT-based radiomics model. *Eur Radiol,* 34**,** 3422-3430 doi: 10.1007/s00330-023-10302-1.
14. Lee, T., Lee, K. H., Lee, J. H., Park, S., Kim, Y. T., Goo, J. M. and Kim, H. (2024c). Prognostication of lung adenocarcinomas using CT-based deep learning of morphological and histopathological features: a retrospective dual-institutional study. *Eur Radiol,* 34**,** 3431-3443 doi: 10.1007/s00330-023-10306-x.
15. Lee, J. K., Han, K., Choi, E., Baek, J., Kim, H. R., Kim, M. D., Kim, H. and Seo, S. K. (2024a). Effect of catheter-directed ethanol sclerotherapy on ovarian reserve in patients with recurrent endometrioma: comparative analysis with primary endometriosis. *Eur Radiol,* 34**,** 3298-3308 doi: 10.1007/s00330-023-10320-z.
16. Li, Y., Zhang, L., Yu, H., Wang, J., Wang, S., Liu, J. and Zheng, Q. (2024). A comprehensive segmentation of chest X-ray improves deep learning-based WHO radiologically confirmed pneumonia diagnosis in children. *Eur Radiol,* 34**,** 3471-3482 doi:10.1007/s00330-023-10367-y.
17. Loch, F. N., Beyer, K., Kreis, M. E., Kamphues, C., Rayya, W., Schineis, C., Jahn, J., Tronser, M., Elsholtz, F. H. J., Hamm, B. and Reiter, R. (2024). Diagnostic performance of Node Reporting and Data System (Node-RADS) for regional lymph node staging of gastric cancer by CT. *Eur Radiol,* 34**,** 3183-3193 doi:10.1007/s00330-023-10352-5.
18. Lv, Y., Ma, X., Zhao, W., Ju, J., Yan, P., Li, S., Xue, Y., Sui, Y., Shao, S., Sun, Q. and Qiu, C. (2024). Association of plaque characteristics with long-term stroke recurrence in patients with intracranial atherosclerotic disease: a 3D high-resolution MRI-based cohort study. *Eur Radiol,* 34**,** 3022-3031 doi: 10.1007/s00330-023-10278-y.
19. Marth, A. A., Feuerriegel, G. C., Marcus, R. P. and Sutter, R. (2024). How accurate is MRI for diagnosing tarsal coalitions? A retrospective diagnostic accuracy study. *Eur* *Radiol,* 34**,** 3493-3502 doi: 10.1007/s00330-023-10304-z.
20. Matsumoto, Y. K., Himoto, Y., Nishio, M., Kikkawa, N., Otani, S., Ito, K., Yamanoi, K., Kato, T., Fujimoto, K., Kurata, Y., Moribata, Y., Yoshida, H., Minamiguchi, S., Mandai, M., Kido, A. and Nakamoto, Y. (2024). Nodal infiltration in endometrial cancer: a prediction model using best subset regression. *Eur Radiol,* 34**,** 3375-3384 doi: 10.1007/s00330-023-10310-1.
21. Nakai, H., Takahashi, H., Adamo, D. A., LeGout, J. D., Kawashima, A., Thomas, J. V., Froemming, A. T., Kuanar, S., Lomas, D. J., Humphreys, M. R., Dora, C. and Takahashi, N. (2024). Decreased prostate MRI cancer detection rate due to moderate to severe susceptibility artifacts from hip prosthesis. *Eur Radiol,* 34**,** 3387-3399 doi: 10.1007/s00330-023-10345-4.
22. Ni, M., He, M., Yang, Y., Wen, X., Zhao, Y., Gao, L., Yan, R., Xu, J., Zhang, Y., Chen, W., Jiang, C., Li, Y., Zhao, Q., Wu, P., Li, C., Qu, J. and Yuan, H. (2024). Application research of AI-assisted compressed sensing technology in MRI scanning of the knee joint: 3D-MRI perspective. *Eur Radiol,* 34**,** 3046-3058 doi: 10.1007/s00330-023-10368-x.
23. Nowak, S., Schneider, H., Layer, Y. C., Theis, M., Biesner, D., Block, W., Wulff, B., Attenberger, U. I., Sifa, R. and Sprinkart, A. M. (2024). Development of image-based decision support systems utilizing information extracted from radiological free-text report databases with text-based transformers. *Eur Radiol,* 34**,** 2895-2904 doi:10.1007/s00330-023-10373-0.
24. Park, C. J., Choi, S. H., Kim, D., Kim, S. B., Han, K., Ahn, S. S., Lee, W. H., Choi, E. C., Keum, K. C. and Kim, J. (2024a). MRI radiomics may predict early tumor recurrence in patients with sinonasal squamous cell carcinoma. *Eur Radiol,* 34**,** 3151-3159 doi:1007/s00330-023-10389-6.
25. Park, J. E., Kim, H. S., Kim, N., Borra, R., Mouridsen, K., Hansen, M. B., Kim, Y. H., Hong, C. K. and Kim, J. H. (2024b). Prediction of pseudoprogression in post-treatment glioblastoma using dynamic susceptibility contrast-derived oxygenation and microvascular transit time heterogeneity measures. *Eur Radiol,* 34**,** 3061-3073 doi:1007/s00330-023-10324-9.
26. Peters, A. A., Solomon, J. B., von Stackelberg, O., Samei, E., Alsaihati, N., Valenzuela, W., Debic, M., Heidt, C., Huber, A. T., Christe, A., Heverhagen, J. T., Kauczor, H. U., Heussel, C. P., Ebner, L. and Wielpütz, M. O. (2024). Influence of CT dose reduction on AI-driven malignancy estimation of incidental pulmonary nodules. *Eur Radiol,* 34**,** 3444-3452 doi: 10.1007/s00330-023-10348-1.
27. Quan, K., Hu, L., Zhang, S., Jin, Y., Wang, D., Luo, J., Ma, Y., Mao, Y. and Zhu, W. (2024). Association of preoperative aneurysmal wall enhancement with relief of chronic headache after surgical clipping of unruptured intracranial aneurysms. *Eur Radiol,* 34**,** 3009-3018 doi: 10.1007/s00330-023-10303-0.
28. Que, Y. T., Chen, Y., Yang, X. Y., Ma, Y. R., Liu, Y. Y., Wen, Z. Q., Lu, B. L., Wu, X. H., Zhang, Z. W., Wu, Y. Z., Yu, S. P. and Yuan, J. P. (2024). MRI-detected tumor deposits in cT3 and cT4 rectal cancer following neoadjuvant chemoradiotherapy. *Eur Radiol,* 34**,** 2963-2973 doi: 10.1007/s00330-023-10261-7.
29. Qu, J., Niu, H., Li, Y., Chen, T., Peng, F., Xia, J., He, X., Xu, B., Chen, X., Li, R., Liu, A., Zhang, X. and Li, C. (2024). A deep learning framework for intracranial aneurysms automatic segmentation and detection on magnetic resonance T1 images. *Eur Radiol,* 34**,** 2838-2848 doi: 10.1007/s00330-023-10295-x.
30. Rigiroli, F., Hamam, O., Kavandi, H., Brook, A., Berkowitz, S., Ahmed, M., Siewert, B. and Brook, O. R. (2024). Routine radiology-pathology concordance evaluation of CT-guided percutaneous lung biopsies increases the number of cancers identified. *Eur* *Radiol,* 34**,** 3271-3283 doi: 10.1007/s00330-023-10353-4.
31. Rosen, S. and Saban, M. (2024). Evaluating the reliability of ChatGPT as a tool for imaging test referral: a comparative study with a clinical decision support system. *Eur Radiol,* 34**,** 2826-2837 doi: 10.1007/s00330-023-10230-0..
32. Sanvito, F., Raymond, C., Cho, N. S., Yao, J., Hagiwara, A., Orpilla, J., Liau, L. M., Everson, R. G., Nghiemphu, P. L., Lai, A., Prins, R., Salamon, N., Cloughesy, T. F. and Ellingson, B. M. (2024). Simultaneous quantification of perfusion, permeability, and leakage effects in brain gliomas using dynamic spin-and-gradient-echo echoplanar imaging MRI. *Eur Radiol,* 34**,** 3087-3101 doi: 10.1007/s00330-023-10215-z.
33. Schwarting, J., Probst, F. A., Griesbauer, M., Robl, T., Burian, E., Wiestler, B., Brunner, T., Malenova, Y., Bumm, C., Folwaczny, M. and Probst, M. (2024). MRI-detected intraosseous bone marrow edema recedes after effective therapy of periodontitis. *Eur* *Radiol,* 34**,** 3115-3122 doi: 10.1007/s00330-023-10327-6.
34. Sim, Y., Kim, M., Kim, J., Lee, S. K., Han, K. and Sohn, B. (2024). Multiparametric MRI-based radiomics model for predicting human papillomavirus status in oropharyngeal squamous cell carcinoma: optimization using oversampling and machine learning techniques. *Eur Radiol,* 34**,** 3102-3112 doi: 10.1007/s00330-023-10338-3.
35. Waelti, S., Skawran, S., Sartoretti, T., Schwyzer, M., Gennari, A. G., Mader, C., Treyer, V., Kellenberger, C. J., Burger, I. A., Hany, T., Maurer, A., Huellner, M. W. and Messerli, M. (2024). A third of the radiotracer dose: two decades of progress in pediatric [(18)F]fluorodeoxyglucose PET/CT and PET/MR imaging. *Eur Radiol,* 34**,** 3252-3259 doi: 10.1007/s00330-023-10319-6.
36. Wang, L., Wang, P., Shao, H., Li, J. and Yang, Q. (2024a). Role of contrast-enhanced mammography in the preoperative detection of ductal carcinoma in situ of the breasts: a comparison with low-energy image and magnetic resonance imaging. *Eur Radiol,* 34**,** 3342-3351 doi: 10.1007/s00330-023-10312-z.
37. Wang, W. X., Gao, Y., Wang, J., Liu, M. X., Gu, H., Yuan, X. S. and Wang, X. M. (2024b). Left ventricular entropy is a novel predictor of major adverse cardiac events (MACE) in patients with coronary atherosclerosis: a multi-center study. *Eur Radiol,* 34**,** 3411-3421 doi: 10.1007/s00330-023-10362-3.
38. Wang, Y., Zhu, Y., Zhang, F., Chen, X., Chen, J., Jiang, W., Chen, S., Yang, J. and Zhang, Q. (2024c). The value of combined ultrasound contrast arthrography and subacromial-subdeltoid bursography for detecting and differentiating the rotator cuff tear subtypes in patients with the uncertain rotator cuff tear. *Eur Radiol,* 34**,** 3503-3512 doi:10.1007/s00330-023-10183-4.
39. Wu, G., Shi, Z., Li, Z., Xie, X., Tang, Q., Zhu, J., Yang, Z., Wang, Y., Wu, J. and Yu, J. (2024a). Study of radiochemotherapy decision-making for young high-risk low-grade glioma patients using a macroscopic and microscopic combined radiomics model. *Eur* *Radiol,* 34**,** 2861-2872 doi: 10.1007/s00330-023-10378-9.
40. Wu, M., Jiang, T., Guo, M., Duan, Y., Zhuo, Z., Weng, J., Xie, C., Sun, J., Li, J., Cheng, D., Liu, X., Du, J., Zhang, X., Zhang, Y. and Liu, Y. (2024b). Amide proton transfer-weighted imaging and derived radiomics in the classification of adult-type diffuse gliomas. *Eur Radiol,* 34**,** 2986-2996 doi: 10.1007/s00330-023-10343-6.
41. Xia, S., Gowda, P., Silva, F. D., Guirguis, M., Ravi, V., Xi, Y. and Chhabra, A. (2024). Comparison between ZOOMit DWI and conventional DWI in the assessment of foot and ankle infection: a prospective study. *Eur Radiol,* 34**,** 3483-3492 doi: 10.1007/s00330-023-10315-w.
42. Xiao, X., Yang, N., Gu, G., Wang, X., Jiang, Z., Li, T., Zhang, X., Ma, L., Zhang, P., Liao, H. and Zhang, L. (2024). Diffusion MRI is valuable in brainstem glioma genotyping with quantitative measurements of white matter tracts. *Eur Radiol,* 34**,** 2921-2933 doi: 10.1007/s00330-023-10377-w.
43. Xie, L. Z., Dou, X. Y., Ge, T. H., Han, X. G., Zhang, Q., Wang, Q. L., Chen, S., He, D. and Tian, W. (2024). Deep learning-based identification of spine growth potential on EOS radiographs. *Eur Radiol,* 34**,** 2849-2860 doi: 10.1007/s00330-023-10308-9.
44. Xu, X., Huang, Y., Liu, Y., Cai, Q., Guo, Y., Wang, H. and Lu, H. (2024). Multiparametric MRI-based VI-RADS: can it predict 1- to 5-year recurrence of bladder cancer? *Eur* *Radiol,* 34**,** 3034-3045 doi: 10.1007/s00330-023-10387-8.
45. Yan, S., Lu, J., Li, Y., Cho, J., Zhang, S., Zhu, W. and Wang, Y. (2024). Spatiotemporal patterns of brain iron-oxygen metabolism in patients with Parkinson's disease. *Eur* *Radiol,* 34**,** 3074-3083 doi: 10.1007/s00330-023-10283-1.
46. Yu, H., Zhao, F., Men, X., Zhu, H., Yan, J., Liu, Z., Liu, Q., Feng, Y., Wang, L., Meng, M., Zhu, Q. and Zhao, X. (2024). Microwave ablation versus laparoscopic resection for hepatocellular carcinoma in patients with clinically significant portal hypertension: a propensity score-matched study of postoperative liver decompensation. *Eur Radiol,* 34**,** 3226-3235 doi: 10.1007/s00330-023-10268-0.
47. Yun, S., Park, J. E., Kim, N., Park, S. Y. and Kim, H. S. (2024). Reducing false positives in deep learning-based brain metastasis detection by using both gradient-echo and spin-echo contrast-enhanced MRI: validation in a multi-center diagnostic cohort. *Eur* *Radiol,* 34**,** 2873-2884 doi: 10.1007/s00330-023-10318-7.
48. Zhang, Y., Chen, J., Yang, C., Dai, Y. and Zeng, M. (2024). Preoperative prediction of microvascular invasion in hepatocellular carcinoma using diffusion-weighted imaging-based habitat imaging. *Eur Radiol,* 34**,** 3215-3225 doi: 10.1007/s00330-023-10339-2.
49. Zhou, Z., Wei, D., Azhe, S., Fu, C., Zhou, X., An, J., Piccini, D., Bastiaansen, J., Guo, Y. and Wen, L. (2024). Self-navigated coronary MR angiography for coronary aneurysm detection in Kawasaki disease at 3T: comparison with conventional diaphragm-navigated coronary MR angiography. *Eur Radiol,* 34**,** 3400-3410 doi: 10.1007/s00330-023-10350-7.
50. Zuiani, C., Mansutti, I., Caronia, G., Linda, A., Londero, V. and Girometti, R. (2024). Added value of the EUSOBI diffusion levels in breast MRI. *Eur Radiol,* 34**,** 3352-3363 doi:1007/s00330-023-10418-4.
